# DNA Methylation Signature of Post-injury Neointimal Cells During Vascular Remodeling in the Rat Balloon Injury Model

**DOI:** 10.4172/2168-9547.1000163

**Published:** 2016-05-18

**Authors:** Jendai Richards, Henry Ato Ogoe, Wenzhi Li, Oguljahan Babayewa, Wei Xu, Tameka Bythwood, Minerva Garcia-Barrios, Li Ma, Qing Song

**Affiliations:** 1 Cardiovascular Research Institute and Department of Medicine, Morehouse School of Medicine, Atlanta, Georgia, USA; 2 Department of Biomedical Informatics, University of Pittsburgh, Pittsburgh, Pennsylvania, USA; 3 4DGenome Inc, Atlanta, Georgia, USA

**Keywords:** Biomarkers, DNA Methylation, Atherosclerosis

## Abstract

Vascular smooth muscle cell (VSMC) accumulation in the neointimal is a common feature in vascular diseases such as atherosclerosis, transplant arteriosclerosis and restenosis. In this study, we isolated the neointimal cells and uninjured residential vascular smooth muscle cells by laser micro dissection and carried out single-cell whole-genome methylation sequencing. We also sequenced the bisulfite converted genome of circulating bone-marrow-derived cells such as peripheral blood mononuclear cells (PBMC) and bone marrow mononuclear cells (BMMC). We found totally 2,360 differential methylation sites (DMS) annotated to 1,127 gene regions. The majority of differentially methylated regions (DMRs) were located in intergenic regions, outside those CpG islands and island shores. Interestingly, exons have less DMRs than promotors and introns, and CpG islands contain more DMRs than islands shores. Pearson correlation analysis showed a clear clustering of neointimal cells with PBMC/BMMC. Gene set enrichment analysis of differentially methylated CpG sites revealed that many genes were important for regulation of VSMC differentiation and stem cell maintenance. In conclusion, our results showed that neointimal cells are more similar to the progenitor cells in methylation profile than the residential VSMCs at the 30^th^ day after the vascular injury.

## Introduction

Vascular smooth muscle cell (VSMC) accumulation in the neointima is a common feature in vascular diseases such as atherosclerosis, transplant arteriosclerosis, and restenosis. In all of the phenomena, one of the characteristic changes is the accumulation of VSMCs within the neointima. The question to be addressed in this work is where the atherosclerotic cells come from, which is very important for the prevention and treatment of atherosclerosis.

Generally, there are two hypothetical sources of the cell origins for atherosclerotic cells, the residential vascular smooth cells [1–9] and the bone-marrow derived progenitor cells [10–13]. This question has not been addressed before because the cell surface markers are not linage specific [14–17] and cell transplants could not escape from immune rejection [7–9]. In recent years, the iPSC works showed that cells keep their memory of origins even after they are reverse differentiated and differentiated, which is embedded in the epigenetics (epigenetic memory) [18–22]. This gives us an opportunity to use epigenetic memory to address the question about atherosclerosis.

## Methods

### Balloon injury model and tissue collection

Wild-type (WT) inbred Sprague Dawley (SD) rats were purchased from Charles River Laboratories. Animal procedures were approved by the Institutional Committee for Use and Care of Laboratory Animals at Morehouse School of Medicine and conform to the Guide for the Care and Use of Laboratory Animals published by US National Institutes of Health. Male Sprague-Dawley rats of 6–10 weeks old were anesthetized with isoflurane. The left carotid artery was exposed via a vertical midline incision and an arteriotomy was performed. The left common carotid artery was injured with an embolectomy balloon catheter (2F Fogarty, Edwards Life Sciences) by inflating to 1.5 atmospheres and retracting to the arteriotomy site three times to assure a good vascular injury [23]. The wound and skin were closed with absorbable and non-absorbable sutures, respectively. Thirty days after injury, rats were euthanized with overexposure to CO_2_, blood was collected from the heart via intra-cardiac puncture; and the injured and normal arteries were exposed, arteries were collected and fixed in 4% formalin. Arteries were embedded in the paraffin block, cut into 10-µm sections, and then mounted onto glass slides and PET FrameSlide (Cat. No. 11505151, Leica Microsystems). The sections were briefly stained by hematoxylin/ eosin and air-dried at room temperature.

### Isolation of neointimal cells by laser microdissection

**Figure 1:**
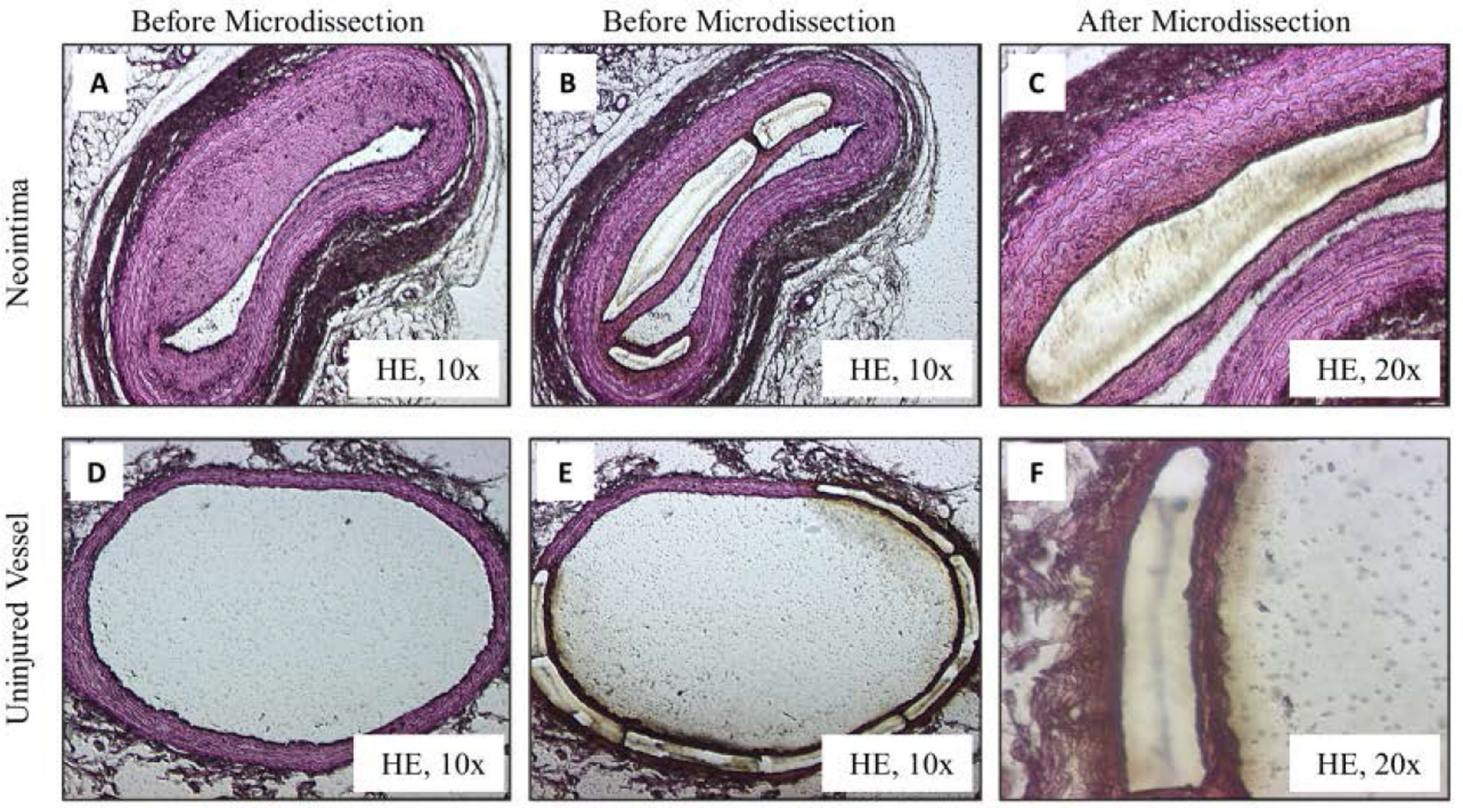
Laser microdissection of normal uninjured arteries and balloon injured arteries **A**. The balloon injured left carotid artery before laser microdissection (10×). There is an intimal thickening and clear border between the normal intimal layer and the newly formed neointima. **B–C**. The balloon injured left carotid artery after microdissection of neointimal cells (10× and 20×). **D**. The normal uninjured right carotid artery before microdissection (10×). **E–F**. The normal uninjured right carotid artery after microdissection (10× and 20×).

A Leica LMD 7000 Laser Microdissection System (Leica Microsystems) was used to isolate cells from the tissue sections. The tissues were visualized under a microscope, target cells were selected and encircled on the computer monitor using a mouse, and then the computer program guided a UV laser (337-nm wavelength) to cut the slide foil with the target cells (Figure 1). The analysis of matched tissue from the same rat avoids confounding effects of the genetic background, previously described as methylation quantitative trait loci [24]. The targeted cells fell into the 0.5-ml thin-wall DNase-free PCR tube caps (BIO plastics) filled with 12 µl DNase/RNase free water (Invitrogen, 75-0024) located beneath the visualized tissue section. The dissection conditions were optimized to obtain a clean, narrow excision of the selected cells: 40-XT objective at power 35 to 45 and speed 3 to 4. Collected samples were centrifuged at full speed (>10,000 ×*g*) for 5 minutes, and stored at –20°C. In the injured arteries, we used the laser to specifically isolate VSMC-like layer in the neointima leaving the endothelial layer intact; we also isolated the resident VSMC population in the medial layer as the control.

### Isolation of BMMC and PBMC

The bone marrow mononuclear cells (BMMCs) and peripheral blood mononuclear cells (PBMCs) were isolated from whole blood and bone marrow aspirate by centrifugation through density gradient centrifugation using Histopaque 1083 (Sigma-Aldrich) as described previously. To isolate PBMCs, about 0.4 ml of blood was collected from each rat using a syringe via aortic puncture. A 21-gauge needle was directed into the thoracic cavity under the xiphoid cartilage while raising the cartilage slightly with the index finger, at approximately a 30–35° angle slightly to the left of the midline. The plunger pulled back on the syringe to create a vacuum. Blood was collected into 8-ml tubes (Becton Dickinson Vacutainer Systems) containing EDTA. Whole blood was centrifuged at 3,500 rpm for 5 min. The entire plasma layer except 5 mm (0.5 mL) was removed. The collection tube was then tilted to a 45° angle and PBMCs was removed from 2 mm above the interface between buffy coat layer and red blood cells (RBC) layer. It was then added to 3.5 mL of PBS in 15 ml centrifuge tube and mixed gently. About 5 mL of dilute buffy coat was then layered on top of 5 mL of HISTOPAQUE 1083 surface in a separate 15 mL centrifuge tube.

To isolate BMMCs, the tibia and femur bones were exposed and cut at the articulatio coxae and talotarsalis, leaving both condyli intact, and keeping the femur and tibia connected. The isolated bone was transferred into a sterile 6-cm Petri dish, filled with 6 mL of cold PBS. The femurs and tibias were then separated by cutting the bone at the articulatio genus, leaving the condyli of both the tibia and femur intact. The ends of the tibia and femur were then cut away. Bone marrow cells were harvested from the male inbred SD rats by flushing tibias and femurs with cold PBS using a sterile 21-gage needle, the samples were passed through a 20.5-gage needle to disperse clumps of cells, and then through a 70-µm filter to further remove bone fragments and cell clumps. Bone marrow cell suspension was transferred to a 15- mL conical tube in cold Magnetic Affinity Cell Sorting (MACS) buffer and spun for 5 min at 675 *g* and 4°C. The supernatant was carefully discarded and the pellet resuspended in 6 mL of MACS buffer at room temperature. About 6 ml of the homogenized bone marrow cell suspension was then layered onto the HISTOPAQUE 1083 surface in a separate 15 ml conical tube.

Histopaque layered buffy coat suspension and bone marrow aspirate suspensions were centrifuged at 400 g for 30 min. Then the upper layer was aspirated, then the tube was tilted at 45° and the mononuclear cell layer was taken and then added to 10 mL of MACS buffer + 2% FBS in 15 mL centrifuge tube and gently mixed. Tubes were spun at 300 *g* for 7 min, and the cell pellet was resuspended in 10ml of MACS buffer.

### Isolation of CD14+ BMMC using MACS

BMMCs were further isolated by CD14 positive using Magnetic Affinity Cell Sorting (MACS). All reagents and supplies for MACS separation were purchased from Miltenyi Biotec, Bergisch-Gladbach, Germany. MACS buffer and instruments were pre-cooled to 4°C prior to use. About 1×10^7^ BMMCs were centrifuged at 300 *g* for 10 min at 4 °C. The supernatant was aspirated and cell pellet was resuspended into 100 µl of cold MACS buffer. Then 2 µl of biotin conjugated rabbit anti-CD14 antibody (Bioss, bs-1192R-Biotin) was added, and incubated at 4°C for 10 min. Cells were washed with 2 ml of cold MACS buffer and centrifuged. The supernatant was aspirated completely and the cell pellet was resuspended in 80 µl of cold MACS buffer. Magnetic labeling of CD14 labeled BMMCs with Anti-Biotin MicroBeads (Miltenyi Biotec, 130-090-485) and positive selection of CD14+ cells was performed following the manufacturer’s instructions. The isolated cell fraction was passed over a new, freshly prepared column according to Miltenyi protocol to increase the purity.

### ViaCount by Guava

The Guava^®^ ViaCount^®^ assay was used to measure the number of BMMCs and PBMCs. During density gradient centrifugation, BMMCs were re-suspended in 10 mL of MACS buffer, and 20 µl of cell suspension was added to 180 µL of ViaCount solution (Guava Technologies 4000-0040) in a 96-well tray removed for ViaCount. The samples were vortexed and incubated, shielded from light, for 8–10 minutes at room temperature.

### Whole genome amplification, library preparation and bisulfite sequencing

Genomic DNA was extracted from PBMCs and BMMCs using the Qiagen DNeasy Blood and Tissue Kit (Qiagen, 69506) according to the manufacturer’s instructions. DNA concentration was measured by the Nanodrop spectrophotometer (Thermo Scientific). The collected neointimal cells and residential VSMCs were directly processed to bisulfite conversion without DNA extraction. The EZ DNA Methylation-Direct™ Kit (ZYMO RESEARCH, D5021) was used to perform bisulfite conversion. For the microdissected samples, 13 µl M-Digestion Buffer and 1 µl Proteinase K were added to 12 µl of sample for digestion. The sample was incubated for 4 h at 50°C. For the BMMCs, we employed 350 ng of genomic DNA for optimized bisulfite conversion. The manufacturer’s protocol for bisulfite conversion was then followed for all samples. After bisulfite conversion, the DNA was bound to a Zymo spin column and desulfonated on the column using M-desulfonation reagent per manufacturer’s instructions. The bisulfite-converted DNA was eluted from the column in 10 µl of water. Bisulfite converted DNA was amplified with The Single Cell Bisulfite Whole Genome Amplification & Library Preparation Kit (Omigenomics) following the manufacturer’s procedure. The products are sequencing-ready library for Illumina sequencer GAII and HiSeq2000. Quantitative PCR was used to measure the concentration of viable sequencing template molecules in the library prior to sequencing. Libraries were quantitated and spiked in 40% Phix prior to loading on an Illumina paired end flow cell (v4), and sequenced on the Illumina Genome Analyzer IIx (Illumina) at a concentration of 11 pM with 101 bp paired read length (paired-end sequencing PE100) as per manufacturer’s instructions. Image analysis and base calling was performed by Illumina Real Time Analysis (RTA) v1.9.35 and output of RTA was demultiplexed and converted to Fastq format with Illumina CASAVA 1.8.2.

### Bioinformatic analysis

We downloaded the rat genome sequence (RGSC 5.0/rn5 assembly) from the University of California Santa Cruz Genome Bioinformatics Site (http://genome.ucsc.edu). Sequence reads were mapped to the rat converted reference genome using the software package BSMAP [25]. Fisher’s exact test was used to evaluate the significance of differential methylation at a single base level. A base was deemed to be differentially methylated if the difference between the mean β value of two sample groups (i.e., case vs control) was >= 0.5 and the adjusted p-value from Fisher’s exact test (false discovery rate correction) was 0.01. The sliding linear model (SLIM) method was used to adjust the observed p-values to q-values [26]. To associate the DMS to a gene region, we computed the distance to the nearest transcription start site (TSS). We did not use cut off (say, TSS distance=µ) to exclude putative genes for downstream analysis such as enrichment analysis with Ingenuity Pathway Analysis (IPA). DMR (differentially methylated regions) analysis was done at single base level. IPA was used for canonical pathway analyses of those validated, differential genes. This bioinformatics tool was used to provide insights into the most involved biological pathways in arterial remodeling based on DNA methylation alterations. Three publicly available rat whole-genome bisulfite sequencing datasets were downloaded from NCBI’s Gene Expression Omnibus (GSE19830). These datasets included liver [27], brain [28], and mammary tissue [29] (GSE50077), mammary gland (GSE40251) and the liver (GSE31571). All SRA files were converted to FASTQ format using the ‘fastq-dump utility in the SRA toolkit. They were analyzed using the same pipeline as described above.

## Results

### Global DNA methylation patterns

We obtained whole-genome bisulfite sequencing data from neointimal cells, residential vascular smooth muscle cells (VSMC), peripheral blood mononuclear cells (PBMCs) and bone marrow mononuclear cells (BMMCs). We generated totally 13,968,170 reads, 13,197,332 reads, 9,067,668 reads, and 11,116,242 reads for BMMCs and PBMCs, neointimal cells, and VSMCs, respectively. We counted the conversion rates at non-CpG cytosines of those uniquely mapped reads. The results are 98.6% (neointimal), 98.4% (VSMCs), 99.3% (BMMCs), and 99.2% (PBMCs). Whole genome methylation sequencing was complemented by publicly available whole-genome bisulfite sequencing data, including mammary tissue, brain and liver.

**Figure 2:**
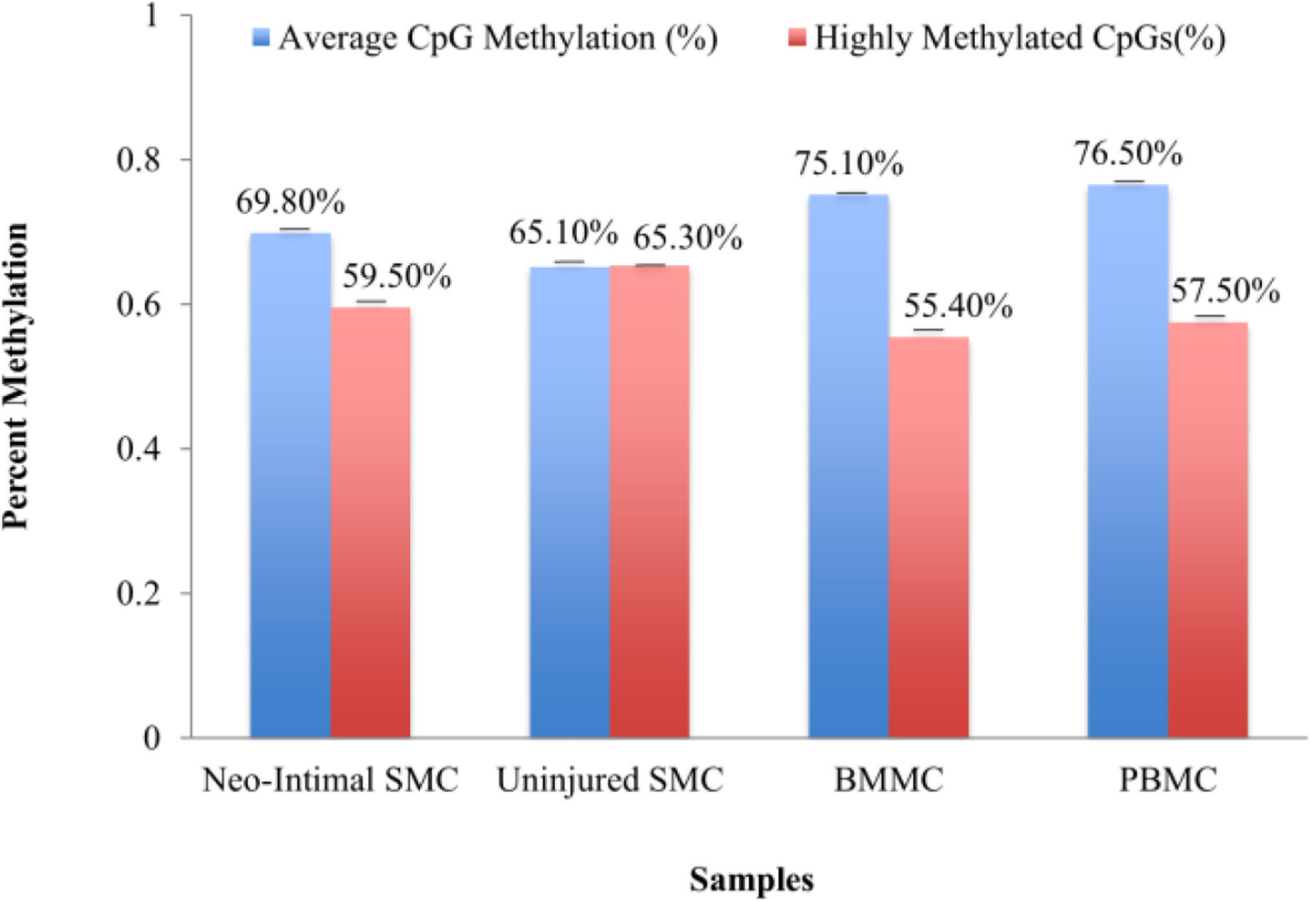
Global CpG methylation analysis Averaged CpG methylation rate and the percentage of highly methylated CpGs are shown for neointimal cells, resident VSMCs, PBMCs and BMMCs. The average methylation shows the ratio of the CpG methylated dinucleotides in each sample to the total number of matched reads. The highly methylated percentage shows the percentage of methylated CpGs with b-values >90%.

**Figure 3:**
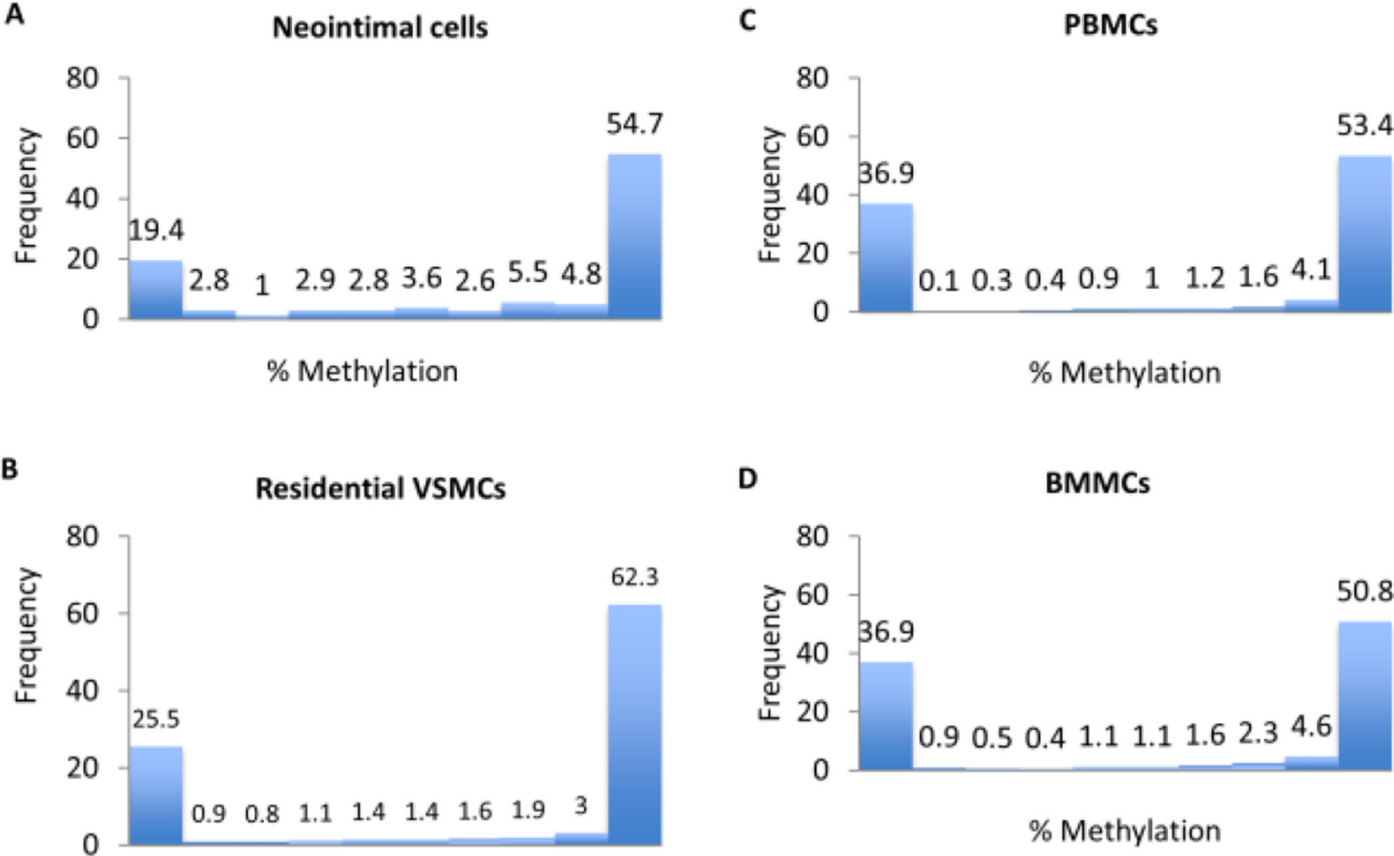
Histograms of methylation percentage per cytosine The distribution of methylation levels (%) across all CpGs is shown for neointimal cells (A), residential VSMCs (B), PBMCs (C) and BMMCs (D). Methylation levels are bimodal denoting that the majority of bases have either high or low methylation.

In general, the neointimal cells showed an overall global methylation percentage similar to the BMMC/PBMC samples (Figure 2). Although the resident VSMCs showed a lower overall CpG methylation rate, they contained more highly (>90%) methylated CpGs (65.3%) than the neointimal cells (59.5%), PBMCs (57.5%) and BMMCs (55.4%). Quantification of average methylation for individual CpGs confirms the normal bimodal distribution of methylation rate in normal cells denoting that the majority of bases are either ‘largely unmethylated’ (<20% of reads showing methylation) or ‘largely methylated’ (>80% of reads) (Figure 3).

**Figure 4:**
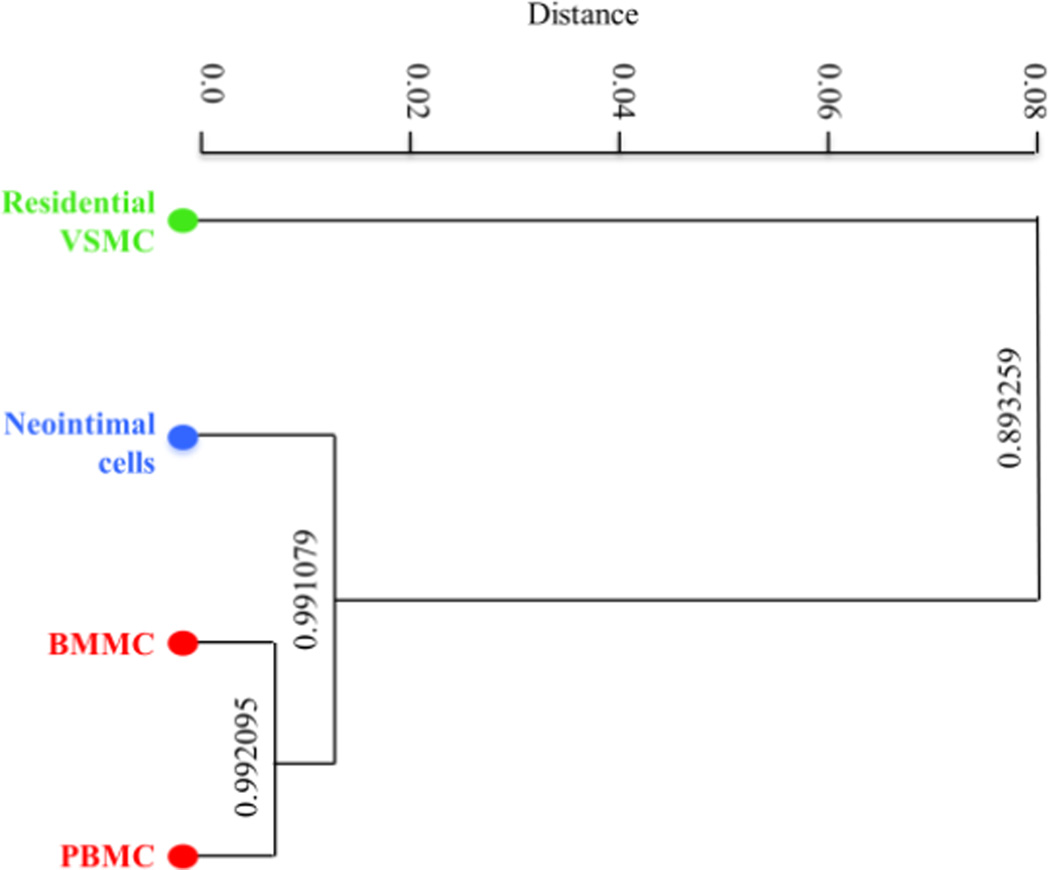
Hierarchical clustering of the neointimal cells, residential VSMCs, PBMCs and BMMCs using Pearson’s correlation distance and “Wards method”. Pair-wise Pearson’s correlation scores are shown in the chart.

We determined which cell types resembles the neointimal cells the most on the methylation profile. We calculated pairwise Pearson correlation coefficients between the neointimal cells and residential VSMC, BMMC and PBMC. The Pearson Correlation coefficients were then used to perform hierarchical clustering of samples based on their molecular signatures. Samples were clustered hierarchically using “correlation” as the distance metric and “Ward’s method” as the agglomeration method in the clustering algorithm. The results showed that the epigenetic signature of neointimal cells were more similar to the BMMC and BMC than to residential VSMC (Figure 4). It is noteworthy that PBMCs and BMMCs were very close even they were obtained from different donors.

### Differential methylation sites

We searched for differential methylation sites (DMS) by comparing the β-values (methylation values ranging from 0.0 to 1.0) in the neointimal cells with residential cells, PBMC and BMMC samples. For calling a CpG site as differentially methylated (single methylation polymorphisms, SMPs), we required a minimum absolute Δβ -value of 0.5 and a false discovery rate (FDR)-adjusted Wilcoxon rank-sum P -value of <0.01 for the difference. The relatively specific SMPs for different cell types are shown in (Supplementary Table 1). In total, 2,360 of the DMS annotated to 1,127 gene regions, among which 52.8% of DMS were hypermethylated in the neointimal samples compared to the resident VSMCs.

### Ingenuity pathway analysis

To explore putative functional roles for the DMS, we performed gene set enrichment analysis using Ingenuity Pathway Analysis tool. Among those genes containing DMS between the neointimal cells and residential VSMCs, 61 DMS were found to be associated with 38 genes, mainly in functional classes, such as cardiovascular system development, cell growth and proliferation, cell death and survival, embryonic development, cellular movement, and skeletal and muscular system development (Supplementary Table 2). The neointimal cells shared 72 DMS on 40 genes with BMMC and PBMC, which are mainly in the systems of cell-mediated immune response, hematological system development and function, embryonic development, inflammatory response.

### Differentially methylated regions (DMRs)

**Figure 5:**
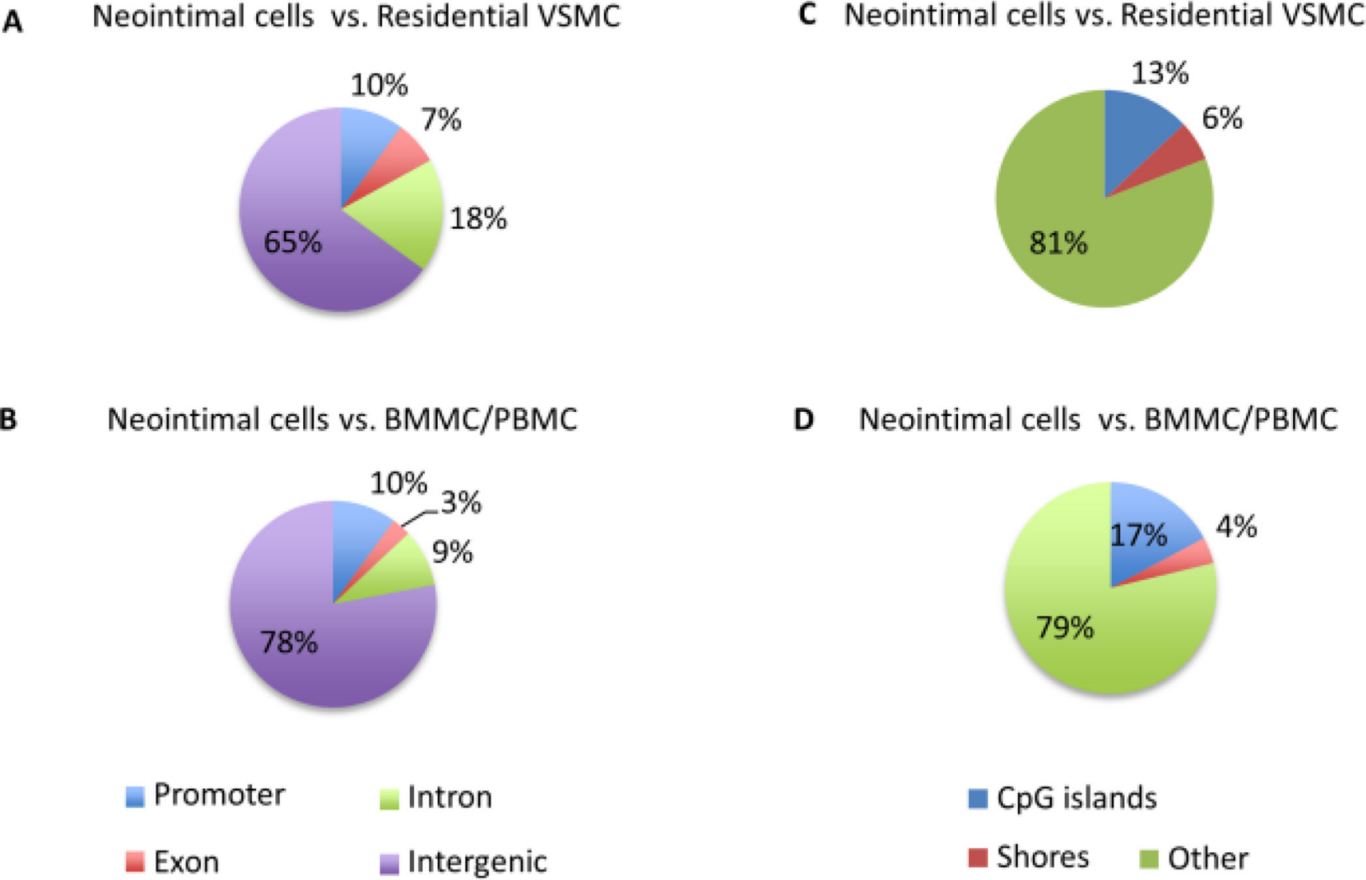
Annotations of different iallymethylate dCpG s ites (a–b) Percentages of differentially methylated CpG sites on promoters, exons, introns and intergenic regions. (c–d) Percentages of differentially methylated CpG sites on CpG islands, CpG island shores (defined as 2kb flanks of CpG islands) and all other regions outside of shores and CpG islands.

Because the PBMCs and BMMCs had very similar DNA methylation profiles, they were combined and analyzed as a single set. We interrogated the non-repetitive part of the genome for regions containing at least five consecutive and consistently differentially methylated CpGs and in which DNA methylation levels of the flanking CpG sites differed significantly between the two samples (Fisher’s exact test, q<0.05 and percent methylation difference larger than 20%). Using these criteria, we identified 662 DMRs between neointimal cells and residential VSMCs, 69 DMRs between neointimal cells and BMMC/PBMC (Supplementary Table 3). DMRs were mostly hyper methylated in the neointimal cells compared with residential VSMCs (Supplementary Table 4). The majority of DMRs were located in intergenic regions, outside those CpG islands and island shores (Figure 5). Interestingly, exons have less DMRs than promotors and introns, and CpG islands contain more DMRs than islands shores (Figure 5).

### Biological relevance of differentially methylated regions

**Figure 6:**
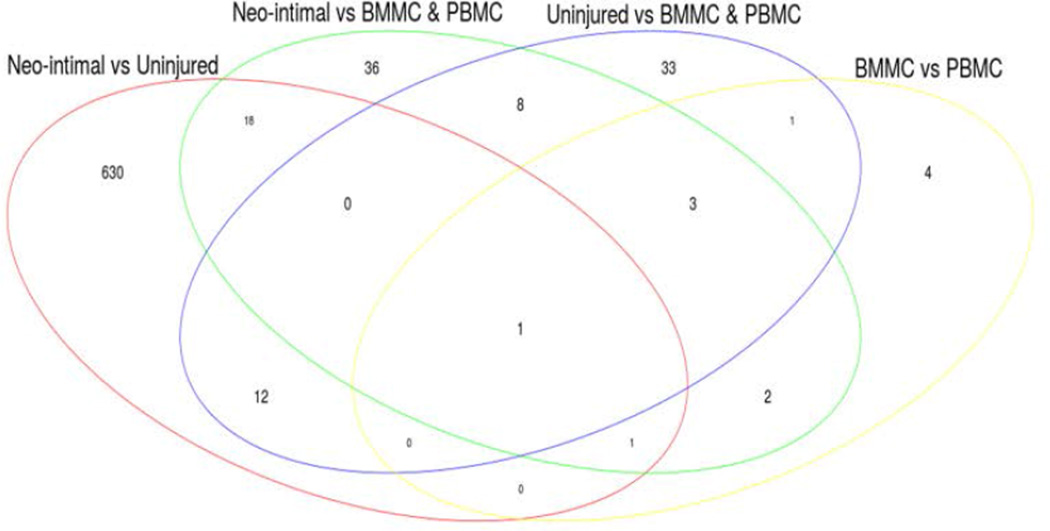
Venn diagram showing differentially methylated regions common and unique to comparisons between different cell lineages.

We used 662 genes corresponding to the DMRs between the neointimal cells vs. residential VSMCs to perform gene set enrichment analysis using Ingenuity Pathway Analysis. We excluded all regions that were also found to be differentially methylated in other pairwise comparisons (Figure 6). Totally, 423 genes were found in the DMRs unique to the comparison between neointimal cells and residential VSMCs (Supplementary Table 5). In this analysis, DMRs that appear in the pairwise comparisons between other cell types in this study were excluded. Furthermore, gene set enrichment analysis showed that the DMRs between neointimal cells and residential VSMCs mainly corresponded to genes in the stem cell pluripotency, cell differentiation, proliferation, and self-renewal categories according to the ranking of statistical significance among various functional categories based on −log (*P* value) (Supplementary Table 6). Other high scoring gene networks include hematologic system development and function, and cardiovascular system development and function. The genes in the DMRs that relate to the regulation of smooth muscle differentiation, VSMC development, and stem cell maintenance and differentiation are provided in (Supplementary Table 7).

## Discussion

In this study, we compared the DNA methylation profiles between neointimal cells, residential VSMCs and bone marrow derived circulating progenitor cells. Our results clearly showed that the atherosclerotic cells keep their epigenetic memory of the bone-marrow derived progenitor cells rather than the residential VSMCs. We believe that these discoveries may guide the development of intervention methods based on the migration of BM derived cells to the site of vascular injury and the proliferation of SMCs after coronary artery bypass surgery or angioplasty. It is known that adult vascular smooth muscle cells (VSMCs) proliferate at a very low rate, exhibit very low synthetic activity, and express a unique set of markers^30^. These cells retain remarkable plasticity and can undergo profound phenotypic transition in response to local environmental changes^14^. When blood vessel is injured, VSMCs dramatically increase their proliferation, migration, and synthetic capacity; however, the high degree of plasticity can also lead to an adverse phenotypic switch and vascular diseases, such as atherosclerosis, restenosis, cancer, and hypertension [30,31] VSMC phenotypic modulation has been a research focus in recent years. It has been discovered that *Klf4* plays a critical role in the phenotypic transitions of VSMCs that have favorable effects in inhibiting plaque pathogenesis [13]; and a microRNA gene (miR-133) also appears to be a key regulator of VSMCs phenotypic switch *in vitro* and *in vivo* [32]. There are some evidences suggesting that neointimal cells mostly derive from vascular cells; although the BM-derived monocytes/macrophages were abundant in the neointima at 7 days after balloon injury, they almost disappeared at 30 days post-surgery [33–38]. Furthermore, a recent study that used *Myh11*-CreER^T2^ ROSA floxed STOP eYFP *Apoe*−/− mice for SMC lineage tracing showed that >80% of SMCs within advanced atherosclerotic lesions are phenotypically modulated and that traditional methods for detecting SMCs based on immunostaining for SMC markers fail to detect >80% of SMC-derived cells within advanced atherosclerotic lesions, indicating that the contribution of SMCs to atherosclerotic plaques has been greatly underestimated by conventional techniques [13].

On the other hand, several studies have suggested that circulating BM derived cells contribute to neointima formation [10,39,40]. In chimeric mice, high-resolution confocal microscopy showed that some BM-derived cells expressed SM-MHC, a specific SMC marker protein [41]; two-photon microscopy techniques observed a trans-differentiation of BM-derived cells into VSMCs [42]. In humans, a combined immunohistochemical and FISH analysis showed a substantial fraction of VSMCs throughout the atherosclerotic vessel wall that were originated from donor bone marrow in the patients receiving sex-mismatched bone marrow transplantation [43]. Recently, it has been reported that blood vessel wall contains a type of stem cells (multipotent vascular stem cells, MVSCs), which can differentiate into SMCs [44]. These studies support that SMCs in the neointima are derived from progenitors. Traditional VSMC markers (such as α-SMA, SM-MHC or calponin) lack lineage-specificity to trace the embryonic origin of cells [30,45–49]. In addition, these markers can change during differentiation and are not suitable for tracing cell origins during trans-differentiations, de-differentiation and differentiations. As reviewed by Owens et al, because a key feature of vascular remodeling is the loss of expression of SMC-selective gene products, such as SM MHC and SM α-actin (SMαA), there are still major ambiguities regarding the definitive identification of altered SMC phenotypes during this process [14]. Green-fluorescent protein [10,50,51] and Y chromosome [37] have been used as markers for tracking the cell origin by distinguishing donor cells from recipient cells in transplantation animal models [49,52], which may be confounded by the immune rejections of transplant models. In the stem cell differentiation and reverse differentiation studies in recent years, it has been suggested that DNA methylation patterns may provide a tool to recognize the cells because they are signatures that contain the memory of re-programmed cells even after the cells switch to another cell type [18–22]. We believe that the comparison of DNA methylation signatures can provide additional insight about the origins of neointimal cells. Our results showed a clustering of the neointimal cells with the PBMCs/BMMCs on DNA methylation patterns. In fact, it has been reported that those unidentified SMC-derived cells also exhibit phenotypes of other cell lineages during the phonotypical SMCs transition within lesions, including macrophages and mesenchymal stem cells (MSCs) but are distinctly different from classical monocytes, macrophages, and dendritic cells [13,53]. Indeed, our study has some caveats. For example, we studied the cells based on their physical positions so we did not further purify the cells based on some cell markers and we did not examine purity. Thus, the collected cells may contain multiple cell types and/or transition states, so our data cannot answer the question where the neointimal cells come from, they may either be differentiated from progenitor cells (circulating or residential) or transitioned from VSMCs. However, our results can answer the question that at a late stage (such as 30th days after injury) the neointimal cells have some signatures of circulating progenitor cells. Another caveat is that our data cannot answer the question if the observation will be similar or different during the remodeling process (earlier than 30^th^ day). The third caveat is that the small sample size, although we isolated 50–100 cells, but they were from one rat, so we cannot eliminate the personal bias from rat to rat. Next caveat is the low coverage, which is similar to an array study, only a small number of CpG sites were studied instead of all CpG sites in the whole-genome, it may cause biases because some CpGs may be more important than others regarding the differentiation or transition during vascular remodeling. In conclusion, we have specifically isolated neointimal cells and compared their methylation signature with the other two hypothesized cell origins; the residential VSMCs and the circulating progenitor cells. To our knowledge this is the first time that laser micro dissection and methylation signatures have been used to address this issue. Although the question about the origination of neointimal cells can only be completely addressed after migration and trans-differentiation are directly observed, our study showed that neointimal cells have a more similar methylation profile to the circulating bone marrow derived cells than the residential VSMCs. The origin of atherosclerotic cells may guide us where our therapeutic strategies should target.

## Supplementary Material

Suppl Table 1

Suppl Table 2

Suppl Table 3

Suppl Table 4

Suppl Table 5

Suppl Table 6

Suppl Table 7
